# Dietary glycemic index and retinal microvasculature in adults: a cross-sectional study

**DOI:** 10.1186/s12937-016-0209-2

**Published:** 2016-10-18

**Authors:** Natalia Sanchez-Aguadero, Rosario Alonso-Dominguez, Jose I. Recio-Rodriguez, Maria C. Patino-Alonso, Manuel A. Gomez-Marcos, Carlos Martin-Cantera, Yolanda Schmolling-Guinovart, Luis Garcia-Ortiz, the EVIDENT II Group

**Affiliations:** 1 Primary Care Research Unit, The Alamedilla Health Center, Castilla and León Health Service (SACYL), Biomedical Research Institute of Salamanca (IBSAL), Spanish Network for Preventive Activities and Health Promotion (redIAPP), Salamanca, Spain; 2 Primary Care Research Unit, The Alamedilla Health Center, Castilla and León Health Service (SACYL), Biomedical Research Institute of Salamanca (IBSAL), Department of Nursing and Physiotherapy, University of Salamanca, Spanish Network for Preventive Activities and Health Promotion (redIAPP), Salamanca, Spain; 3 Department of Statistics, University of Salamanca, Biomedical Research Institute of Salamanca (IBSAL), Spanish Network for Preventive Activities and Health Promotion (redIAPP), Salamanca, Spain; 4 Primary Care Research Unit, The Alamedilla Health Center, Castilla and León Health Service (SACYL), Biomedical Research Institute of Salamanca (IBSAL), Department of Medicine, University of Salamanca, Spanish Network for Preventive Activities and Health Promotion (redIAPP), Salamanca, Spain; 5 Passeig de Sant Joan Health Center, Catalan Health Service, Spanish Network for Preventive Activities and Health Promotion (redIAPP), Barcelona, Spain; 6 Río Tajo Health Center, Castilla-La Mancha Health Service, University of Castilla-La Mancha, Spanish Network for Preventive Activities and Health Promotion (redIAPP), Talavera de la Reina, Spain; 7 Primary Care Research Unit, The Alamedilla Health Center, Castilla and León Health Service (SACYL), Biomedical Research Institute of Salamanca (IBSAL), Department of Biomedical and Diagnostic Sciences, University of Salamanca, Spanish Network for Preventive Activities and Health Promotion (redIAPP), Salamanca, Spain; 8 Primary care Research Unit, The Alamedilla Health Center, Avda. Comuneros N° 27, 37003 Salamanca, Spain

**Keywords:** Glycemic index, Retinal vessels, Carbohydrates, Microcirculation

## Abstract

**Objective:**

To analyze the relationship between dietary glycemic index (GI) and retinal microvasculature in adults.

**Methods:**

This was a cross-sectional study of 300 subjects from the EVIDENT II study. Dietary GI was calculated using a validated, semi-quantitative food frequency questionnaire. Retinal photographs were digitized, temporal vessels were measured in an area 0.5–1 disc diameter from the optic disc and arteriolar-venular index (AVI) was estimated with semi-automated software.

**Results:**

AVI showed a significant difference between the tertiles of GI, after adjusting for potential confounders. The lowest AVI values were observed among subjects in the highest tertile of GI, whereas the greatest were found among those in the lowest tertile (estimated marginal mean of 0.738 vs. 0.768, *p* = 0.014).

**Conclusions:**

In adults, high dietary GI implies lowering AVI values regardless of age, gender and other confounding variables.

**Trial registration:**

Clinical Trials.gov Identifier: NCT02016014. Registered 9 December 2013.

## Background

The glycemic index (GI) represents the relative rate at which blood glucose levels rise after consuming 1 g of a carbohydrate-containing food as compared to pure glucose [1]. High GI diets are associated with an increased risk of cardiovascular diseases (CVD) [2].

Accumulating evidence suggests that the development of CVD such as stroke could be predicted by retinal microvascular changes [3]. Retinal microcirculation has been linked to GI in a few studies [4, 5]. This association might be mediated for oxidative stress or inflammation [6–8].

The purpose of this study was to analyze the relationship of dietary GI with retinal microvasculature in a sample of adults.

## Methods

A cross-sectional study was conducted with 300 subjects, as a sub-analysis of the EVIDENT II trial [9]. The recruitment and data collection period was from January 2014 to May 2015.

Procedures for collecting sociodemographic and clinical data, obtaining analytical parameters and performing office blood pressure and anthropometric measurements have been reported in a prior publication [9].

A food frequency questionnaire (FFQ) validated for Spain [10] was used to calculate composition of carbohydrates, proteins and fats, total calories and GI for each participant’s diet. In the FFQ, subjects indicated the frequency of intake of a number of food items during the previous year, divided into nine categories of consuming, ranging from never to more than six times per day. The daily dietary GI for each subject was computed dividing his dietary glycemic load (GL) by his total carbohydrate intake per day. Dietary GL was obtained by summing GL of each consumed food (corresponding GI x carbohydrate content per serving x average number of servings per day) [1].

Retinography was performed using a Topcon TRC NW 200 non-mydriatic retinal camera (Topcon Europe B.C., Capelle a/d Ijssel, The Netherlands), obtaining nasal and temporal images centered on the disc. The nasal image with the centered disc was loaded into an arteriolar-venular index (AVI) calculator developed for us (Ciclorisk SL, Salamanca, Spain; registry no. 00/2011/589), whose validation has been published elsewhere [11]. This software automatically recognizes the disc and draws two external concentric circles which delimit area A, between 0 and 0.5 disc diameters from the optic disc margin; and area B, between 0.5 and 1 disc diameters from the margin. It finally estimates the mean caliber of venules and arterioles circulating through area B in micrometers (μm), and summarizes them as a ratio, AVI. An AVI of 1.0 suggests that arteriolar diameters are on average the same as venular diameters in that eye; whereas a smaller AVI suggests narrower arterioles [12]. We used the pairs of main vessels in the upper and lower temporal quadrants, rejecting the rest, to improve the reliability and increase efficiency of the process.

Continuous variables were expressed as the mean ± standard deviation, and qualitative variables as frequency distributions. We used a multivariate analysis based on the analysis of covariance (ANCOVA) method, to compare the retinal microvasculature variables between tertiles of GI. The model was adjusted for age, gender, total energy intake, body mass index (BMI), systolic blood pressure (SBP) and medical treatment (antihypertensive, antidiabetic and lipid-lowering drugs). IBM SPSS Statistics for Windows version 23.0 (Armonk, NY: IBM Corp) was used. A value of *p* < 0.05 was considered statistically significant.

## Results

The mean age of the sample group was 51.6 years (64.3 % females), of whom 77 (25.7 %) were hypertensives, 13 (4.3 %) were type 2 diabetics, 84 (28 %) had dyslipidemia, 73 (24.3 %) had a BMI higher than 30 kg/m^2^ and 83 (27.7 %) were smokers. The proportion of patients treated with antihypertensive, antidiabetic and lipid-lowering agents was 20.7 %, 4.3 % and 14 %, respectively. The mean blood pressure (BP) was 121/74 mmHg, with a mean BMI of 27.3 Kg/m^2^ and a waist circumference of 92.9 cm. The median values of total cholesterol, triglycerides, serum glucose and HbA1c were 200.5 mg/dl, 99.7 mg/dl, 85.3 mg/dl and 5.4 %, respectively. The average total energy intake was 2547.9 ± 757.0 Kcal/day with a mean carbohydrates consumption of 274.7 ± 97.7 g/day and an overall GI of 47.8 ± 5.5. The mean AVI, calculated from a retinal arteriolar caliber of 100.8 ± 11.4 μm and a venular caliber of 134.6 ± 14.5 μm, was 0.76 ± 0.08. Table 1.

**Table 1:** Baseline characteristics

	Mean or n/SD or %
Age	51.6 (10.4)
Sex (% females)	193 (64.3)
Hypertension (n, %)	77 (25.7)
Type 2 Diabetes (n, %)	13 (4.3)
Dyslipidemia (n, %)	84 (28.0)
Obesity, BMI > 30 (n, %)	73 (24.3)
Smoking (n, %)	83 (27.7)
Antihypertensive drugs (n, %)	62 (20.7)
Antidiabetic drugs (n, %)	13 (4.3)
Lipid-lowering drugs (n, %)	42 (14.0)
Systolic blood pressure (mmHg)	121.3 (16.4)
Diastolic blood pressure (mmHg)	74.3 (10.2)
Heart rate (bpm)	67.7 (10.6)
BMI (Kg/m^2^)	27.3 (4.6)
Waist circumference (cm)	92.9 (12.4)
Serum glucose (mg/dl)	85.3 (11.4)
HbA1c (%)	5.4 (0.4)
Total cholesterol (mg/dl)	200.5 (32.1)
Triglycerides (mg/dl)	99.7 (46.6)
Total energy (Kcal/day)	2547.9 (757.0)
Total fat (g/day)	105.7 (36.5)
Protein (g/day)	110.0 (28.9)
Carbohydrates (g/day)	274.7 (97.7)
GI (%)	47.8 (5.5)
Arteriolar caliber (μm)	100.8 (11.4)
Venular caliber (μm)	134.6 (14.5)
AVI	0.76 (0.08)

Data for qualitative variables are expressed as n (%) and quantitative variables as mean ± standard deviation

*BMI* body mass index, *GI* glycemic index, *AVI* retinal arteriolar-venular index

**Fig. 1:**
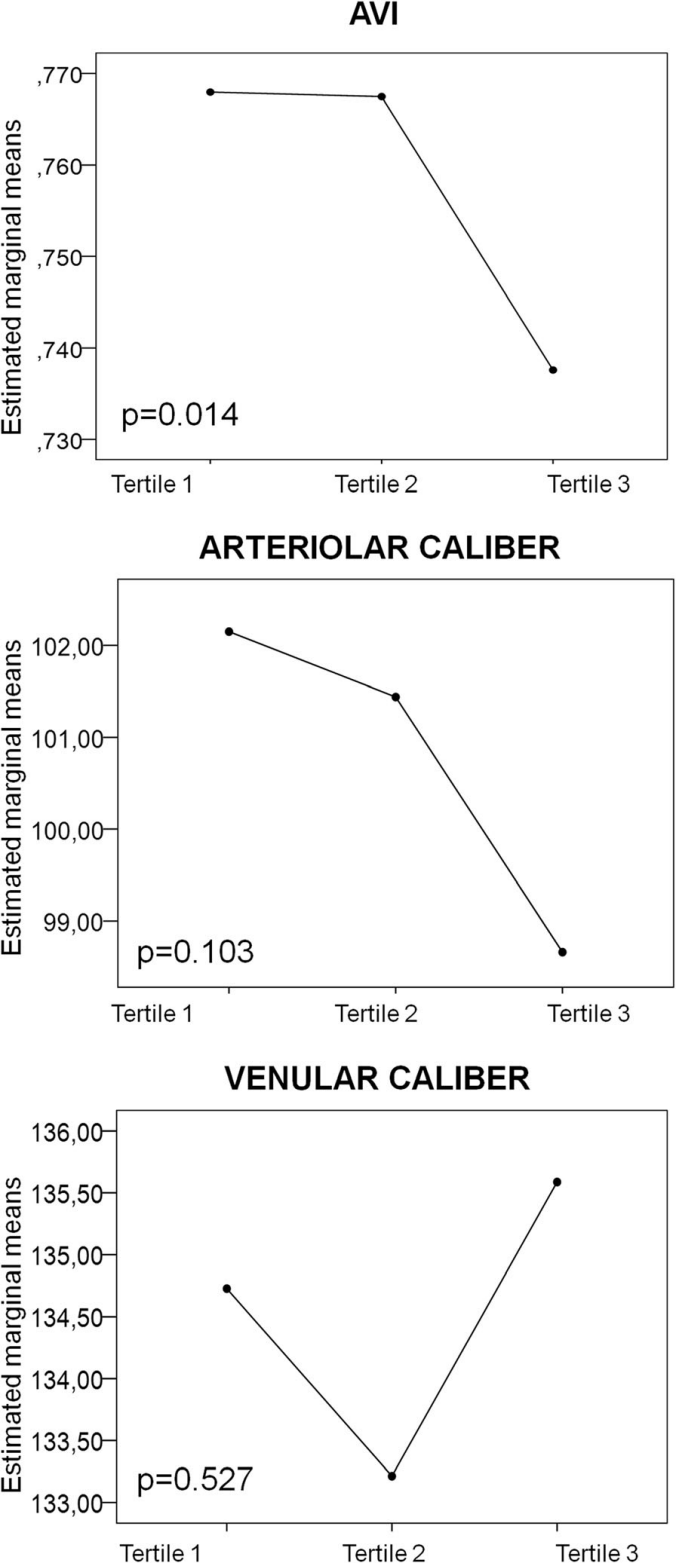
Multivariate analysis. Retinal arteriolar-venular index (AVI), retinal arteriolar caliber and retinal venular caliber by tertiles of glycemic index (GI). Model adjusted for age, gender, total energy intake, body mass index (BMI), systolic blood pressure (SBP), antihypertensive drugs, antidiabetic drugs and lipid-lowering drugs. Tertiles (T) GI: T1 (Lowest through 45.98); T2 (45.98 through 50.52); T3 (50.52 through Highest). AVI differences by tertiles of GI: *p* = 0.033 between T1 and T3, *p* = 0.031 between T2 and T3. Post-hoc contrasts were performed by the Bonferroni test

In the multivariate analysis, AVI showed a significant difference between tertiles of GI, after adjusting for potential confounders. There were no differences in the case of the retinal arteriolar or venular caliber. As illustrated by Fig. 1, lower AVI values were observed among individuals in the third tertile of GI (i.e., the highest) while the greatest were found among those in the first tertile of GI (i.e., the lowest) (an estimated marginal mean of 0.738 vs. 0.768, *p* = 0.014). Furthermore, a tendency towards reduction of the arteriolar caliber among subjects of the highest tertiles of GI was revealed. A subanalysis was performed in hypertensive (*n* = 77) and dyslipidemic individuals (*n* = 84), using the same model adjustment than in the overall sample. We found the lowest AVI in the third tertile of GI (the highest) in dyslipidemic subjects. However the differences did not reach statistical significance (*p* = 0.085). Also, we have not found significant differences in hypertensive subjects (*p* = 0.500).

## Discussion

The results of our study show that a higher dietary GI implies lower AVI values in a sample of adults, after multivariable adjustment. This suggests that the protective effect from low GI food consumption against vascular disease could partly explain the changes in retinal microvasculature. To our knowledge, high GI diets had never been linked to lower AVI values in the adult population. It has been postulated that a smaller AVI reflects generalized arteriolar narrowing and predicts the risk of CVD [13]. These data highlight the potential role of low GI diets in the intervention strategies for reducing cardiovascular risk (CVR).

We conducted an analysis of covariance (ANCOVA) to control the effect of certain confounding variables that was not possible to control due to the type of the experimental design. Therefore, we believe that the results are consistent and independent of the influence of age, gender and other variables used in the model. Our findings indicate that dietary GI has a greater influence on retinal arteriolar-venular ratio than on arteriole or venule caliber separately. These data are similar to those collected by Lim et al. [14], who reported no significant associations between the caliber of retinal vessels and carbohydrate or sugar intake in schoolchildren. In contrast, a later study of 2,353 12-y-old children recorded, in girls, a narrowing of the retinal arterioles and a widening of its venules with increasing dietary GI [4]. Previously, Kaushik et al. [5] had found an association between higher dietary GI and wider retinal venular caliber in person 50 years and older. A possible explanation for this inconsistency with our results might be the use of different methods for assessing the retinal vessels caliber. However, in coincidence with our study, these authors observed a trend to a decreased retinal arteriolar caliber as dietary GI increased, despite their larger sample.

The limitations of our study include the fact that the cross-sectional design prevents the establishment of causal relationships between dietary GI and retinal microvasculature, GI was estimated using a self-administered questionnaire that had not been specifically developed to elucidate it and retinal microvasculature was assessed from two vessels (an arteriole and a venule) as described above.

## Conclusions

In conclusion, a high dietary GI implies lowering AVI values in adults regardless of age, gender and other confounding variables. Further longitudinal studies would be needed to confirm the observed relationship.

## Abbreviations

ANCOVA: Analysis of covariance
AVI: Arteriolar-venular index
BMI: Body mass index
BP: Blood pressure
CVD: Cardiovascular diseases
CVR: Cardiovascular risk
EVIDENT: Lifestyles and vascular aging
FFQ: Food frequency questionnaire
GI: Glycemic index
GL: Glycemic load
SBP: Systolic blood pressure
μm: Micrometers
